# Case Report: Primary ovarian pulmonary-type small cell neuroendocrine carcinoma mimicking benign adnexal torsion: a diagnostic challenge

**DOI:** 10.3389/fmed.2026.1941804

**Published:** 2026-09-14

**Authors:** Sijia Xu, Pinqiu Lv, Na Li, Bin Wang, Xuan Du, Zhitao Yao

**Affiliations:** 1 Department of Gynecology, The First Affiliated Hospital of Zhejiang Chinese Medical University, Hangzhou, China; 2 Department of Obstetrics and Gynecology, Quzhou Municipal Hospital of Traditional Chinese Medicine, Quzhou, Zhejiang, China

**Keywords:** acute abdomen, adnexal torsion, hemoperitoneum, neuroendocrine carcinoma, small cell carcinoma of the ovary, pulmonary type

## Abstract

**Background:**

Small cell carcinoma of the ovary, pulmonary type (SCCOPT), is a rare and aggressive ovarian neuroendocrine carcinoma. Because its clinical and radiological features overlap with benign adnexal conditions and metastatic neuroendocrine tumors, preoperative diagnosis remains challenging. Here, we report a rare case of SCCOPT presenting as acute abdomen associated with adnexal torsion, tumor rupture, and hemoperitoneum, which complicated recognition of the underlying malignancy.

**Case presentation:**

A woman in her early 60s presented with acute lower abdominal pain, abdominal distension, and syncope. Pelvic magnetic resonance imaging revealed a complex cystic-solid adnexal mass. Emergency laparoscopy demonstrated left adnexal torsion, tumor rupture, active bleeding, and approximately 550 mL of hemoperitoneum. Histopathological examination revealed a high-grade small cell neuroendocrine carcinoma. Immunohistochemical analysis supported the diagnosis of SCCOPT, showing positivity for synaptophysin, chromogranin A, and CD56, with negative TTF-1 expression, loss of Rb expression, and retained SMARCA4 expression. Postoperative evaluation, including PET-CT, gastroscopy, and colonoscopy, showed no evidence of an extra-ovarian primary tumor. Tumor–blood sequencing identified an HRD-negative/BRCA-wild-type profile and a somatic TP53 mutation, further supporting the high-grade neuroendocrine phenotype. The patient underwent surgical staging followed by six cycles of carboplatin plus etoposide chemotherapy and remained without clinically evident recurrence at the latest follow-up.

**Conclusion:**

SCCOPT may initially present as an acute gynecological emergency and mimic benign adnexal torsion, potentially delaying recognition of malignancy. Accurate diagnosis requires integration of clinical presentation, imaging findings, histopathology, immunophenotyping, molecular alterations, and exclusion of extra-ovarian primary sites. This case emphasizes the importance of considering ovarian malignancy, including SCCOPT, in postmenopausal women presenting with acute adnexal events.

## 1 Introduction

Ovarian neuroendocrine neoplasms (O-NENs) are rare tumors, accounting for less than 1% of ovarian malignancies (1). Small cell carcinoma of the ovary, pulmonary type (SCCOPT), is an exceptionally rare and aggressive subtype of ovarian neuroendocrine carcinoma, with only a limited number of reported cases and generally poor outcomes (2–4).

The current WHO classification recognizes small cell carcinoma of the ovary, hypercalcemic type (SCCOHT), as a distinct SMARCA4-deficient ovarian tumor entity rather than an ovarian neuroendocrine carcinoma (5). SCCOHT typically occurs in younger women and is characterized by SMARCA4 inactivation, whereas SCCOPT is an exceptionally rare ovarian neuroendocrine carcinoma that predominantly occurs in older, postmenopausal women (4, 6). Histologically, SCCOPT resembles small cell lung carcinoma (SCLC) (7, 8). Diagnosis requires exclusion of metastatic extra-ovarian neuroendocrine carcinoma and distinction from other ovarian small cell tumors, particularly SCCOHT (9).

Because SCCOPT lacks specific clinical and radiological features, preoperative diagnosis remains difficult. Most patients present with nonspecific symptoms such as abdominal distension, pelvic pain, or vaginal bleeding (7). In contrast, acute abdomen caused by adnexal torsion, tumor rupture, and hemoperitoneum is uncommon in ovarian malignancies. Malignant tumors are generally considered less prone to torsion because adhesions and infiltrative growth may limit ovarian mobility (10, 11). Thus, the coexistence of torsion, rupture, and hemoperitoneum may obscure the malignant nature of the lesion and delay recognition of the underlying tumor. Although acute abdomen is a well-recognized presentation of benign adnexal disorders, it is rarely reported as the initial manifestation of SCCOPT. Only a limited number of SCCOPT cases presenting with acute abdomen have been described (12, 13). This unusual presentation represents a diagnostic pitfall, as emergency clinical scenarios may divert attention from a possible ovarian malignancy.

Given its rarity, no standardized treatment has been established for SCCOPT. Current management is largely based on strategies used for SCLC and other high-grade neuroendocrine carcinomas, with surgery followed by platinum-based chemotherapy commonly adopted, although the optimal regimen remains uncertain.

Herein, we report a case of primary ovarian SCCOPT presenting as acute abdomen caused by adnexal torsion, tumor rupture, and hemoperitoneum. This case emphasizes that ovarian malignancy should be considered even when the initial presentation resembles a benign gynecologic emergency. The diagnosis was established through integrated evaluation of imaging findings, pathological examination, molecular profiling, and comprehensive exclusion of an extra-ovarian primary tumor.

## 2 Case presentation

### 2.1 Patient history and clinical findings

A 61-year-old postmenopausal woman (G1P1) presented to the emergency department on January 30, 2026, with acute abdominal pain, abdominal distension, and one episode of syncope. She reported a 1-week history of intermittent lower abdominal discomfort, which had significantly worsened over the preceding 5 h. She denied abnormal vaginal bleeding, vaginal discharge, fever, gastrointestinal symptoms, or urinary complaints. Her medical history was unremarkable, with no previous abdominal or pelvic surgery, gynecological disease, or malignancy. There was no personal or family history of ovarian, breast, colorectal, or other malignancies.

On admission, the patient was hemodynamically stable. Physical examination revealed mild abdominal distension and lower abdominal tenderness without rebound tenderness or guarding. Pelvic examination identified a tender left adnexal mass measuring approximately 7 × 8 cm, whereas the right adnexa was unremarkable. The clinical course is summarized in Figure 1.

**Figure 1:**
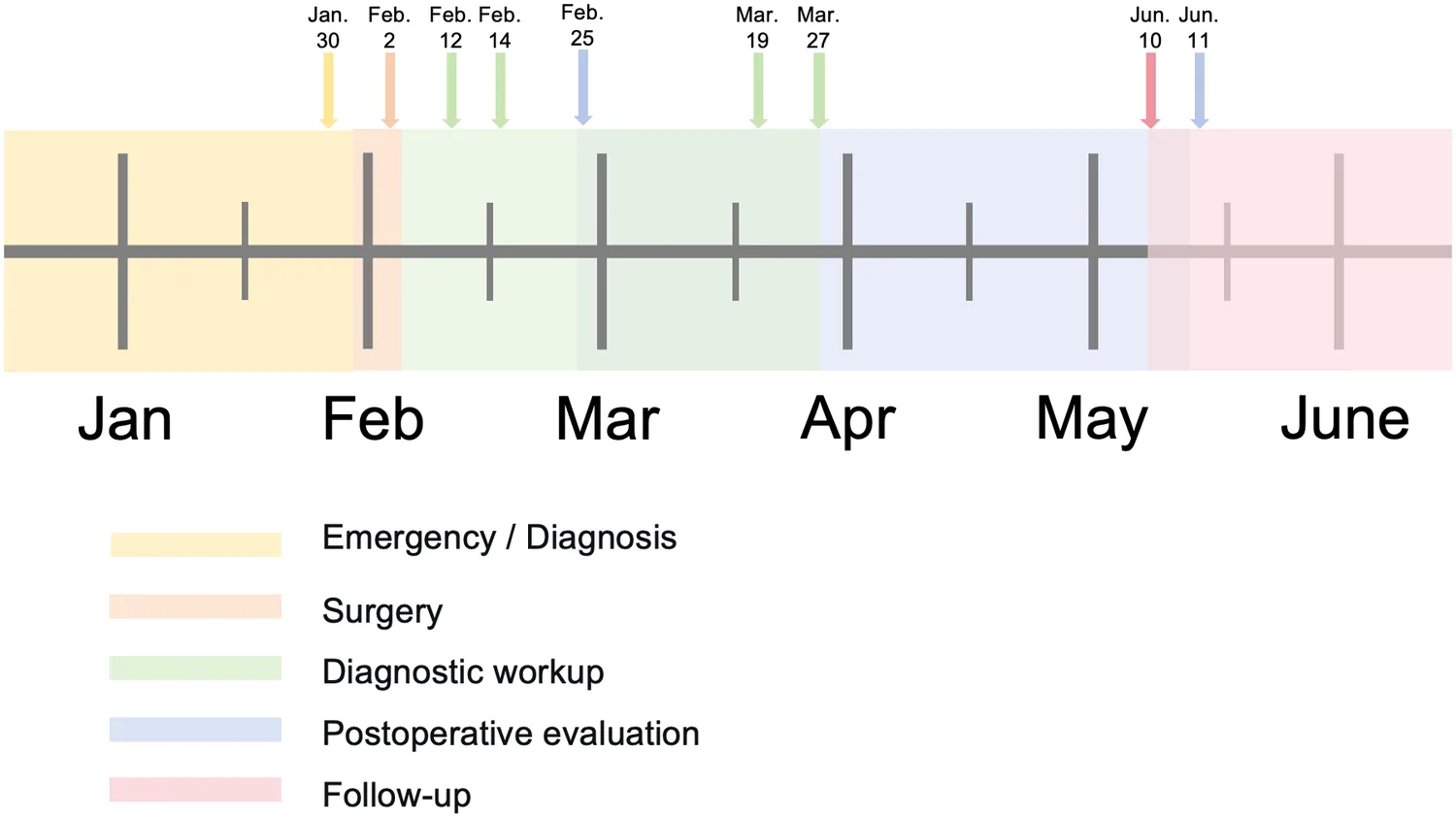
Clinical timeline of diagnosis, treatment, and follow-up. The clinical course of the patient from emergency presentation to postoperative follow-up from January to June 2026 is summarized. Key clinical events included emergency presentation with acute abdomen (January 30), laparoscopic staging surgery (February 2), postoperative PET-CT evaluation (February 12), histopathological and immunohistochemical confirmation of SCCOPT (February 14), initiation of adjuvant carboplatin plus etoposide chemotherapy (February 25), gastroscopy and colonoscopy excluding extra-ovarian primary malignancy (March 19), paired tumor–blood next-generation sequencing (March 27), completion of six cycles of chemotherapy (June 10), and contrast-enhanced CT follow-up showing no evidence of recurrence or distant metastasis (June 11). Colored bars indicate the major clinical phases: Emergency/Diagnosis, Surgery, Diagnostic workup, Postoperative evaluation, and Follow-up.

### 2.2 Laboratory and imaging findings

Initial laboratory evaluation showed no evidence of anemia. Serum CA-125 and HE4 levels were mildly elevated, while other tumor markers were within normal limits.

Transvaginal ultrasonography revealed a cystic-solid mass in the left adnexal region measuring approximately 8.2 × 6.0 cm, with internal vascularity. Moderate pelvic free fluid was observed, with a maximal depth of approximately 5.9 cm. Given the acute abdominal symptoms and episode of syncope, intra-abdominal bleeding was suspected, and hemoperitoneum was subsequently confirmed during surgery (Figures 2A,B).

**Figure 2:**
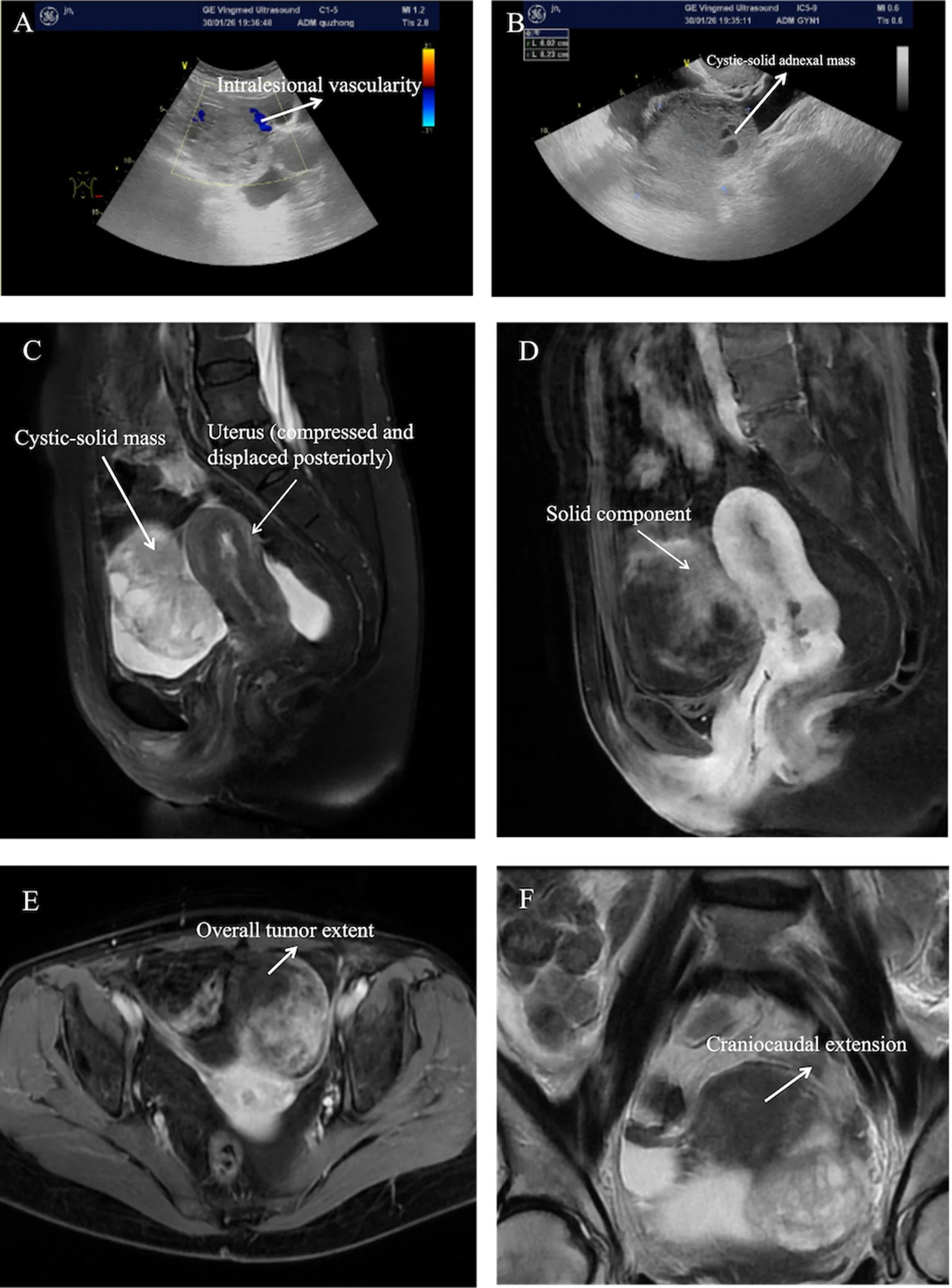
Clinical presentation and imaging findings. **(A,B)** Transvaginal ultrasonography revealed a left adnexal cystic-solid mass with internal vascularity within the solid component. The lesion measured approximately 8.2 × 6.0 cm and was accompanied by pelvic free fluid. **(C–F)** Pelvic magnetic resonance imaging demonstrated a multiloculated cystic-solid mass arising from the left adnexa. The lesion showed heterogeneous signal intensity, restricted diffusion of the solid component, and marked enhancement of the cyst wall, septa, and solid portions, producing a honeycomb-like appearance.

Contrast-enhanced pelvic magnetic resonance imaging (MRI) demonstrated a multiloculated cystic-solid mass arising from the left adnexa, measuring approximately 64 × 63 × 90 mm. The lesion showed multiple cystic components and a relatively well-defined margin. The solid components exhibited restricted diffusion with high signal intensity on diffusion-weighted imaging. After contrast administration, the cyst wall, septa, and solid components showed marked enhancement, resulting in a honeycomb-like appearance. Based on the multiloculated cystic-solid morphology and enhancement pattern, an adult granulosa cell tumor was considered the leading differential diagnosis; however, ovarian malignancy could not be excluded (Figures 2C–F).

### 2.3 Surgical findings and management

Given the acute abdominal presentation, adnexal mass, and suspected hemoperitoneum, urgent surgical intervention was indicated. As the patient remained hemodynamically stable without evidence of ongoing massive hemorrhage, pelvic MRI was performed before surgery to further characterize the lesion and facilitate surgical planning.

Exploratory laparoscopy was performed on February 2, 2026. Upon entry into the peritoneal cavity, approximately 550 mL of hemoperitoneum was identified and evacuated. The left ovarian tumor was found to be torsed approximately 180 at the pedicle, with evidence of preoperative capsular rupture and active bleeding (Figures 3A,B). The uterus and contralateral adnexa were grossly unremarkable, and no obvious extra-ovarian lesions were identified within the abdominal or pelvic cavity.

**Figure 3:**
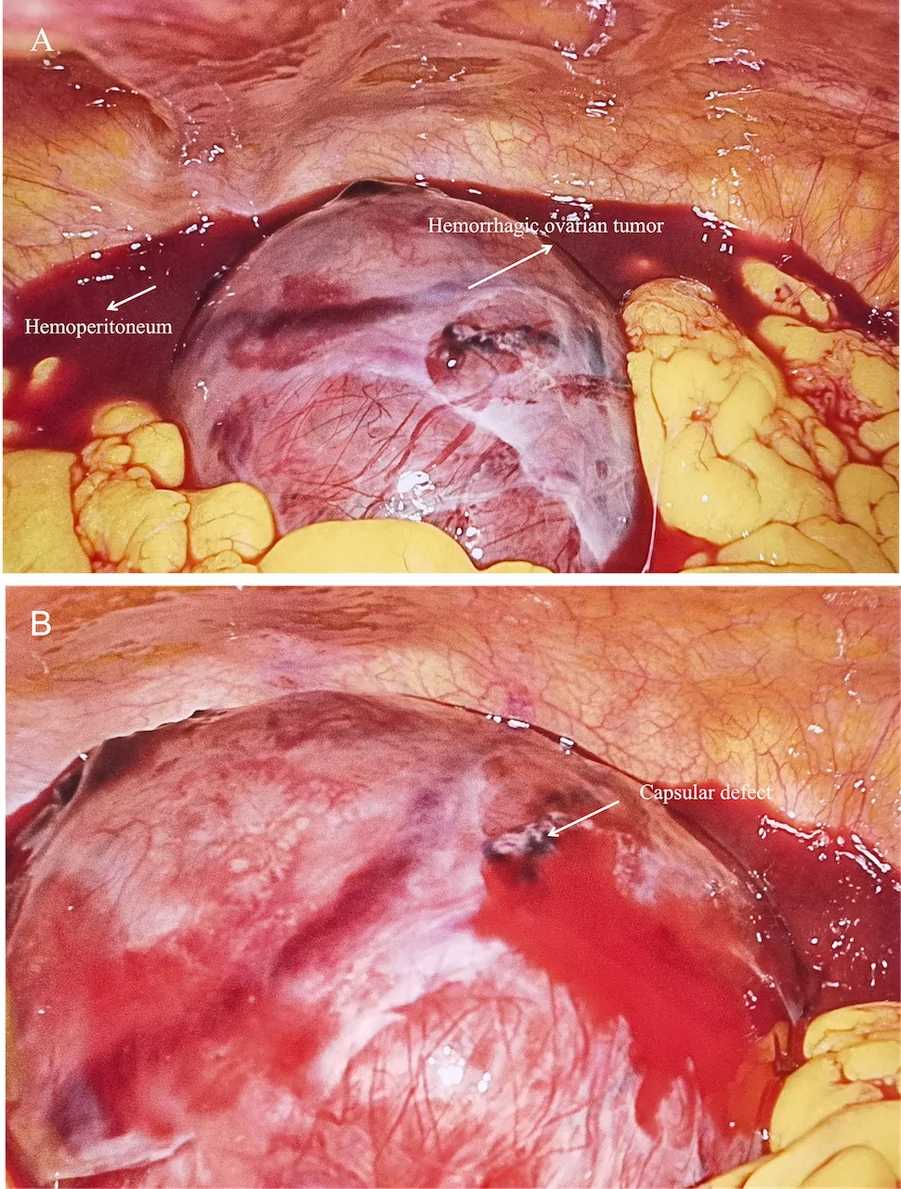
Intraoperative laparoscopic findings. **(A)** Laparoscopic exploration revealed an enlarged hemorrhagic left ovarian tumor associated with hemoperitoneum. **(B)** A capsular defect with active bleeding was identified on the tumor surface, consistent with preoperative tumor rupture.

Peritoneal fluid and lavage cytology showed no malignant cells. Intraoperative frozen section examination suggested a malignant ovarian tumor. Therefore, comprehensive laparoscopic staging surgery was performed, including total hysterectomy, bilateral salpingo-oophorectomy, omentectomy, bilateral pelvic lymphadenectomy, peritoneal lavage, and systematic exploration of the abdominal and pelvic cavity.

The procedure was completed laparoscopically without intraoperative complications. Estimated blood loss was approximately 200 mL, and no allogeneic blood transfusion was required. According to the FIGO staging system, the tumor was classified as stage IC2 based on preoperative tumor rupture. Peritoneal cytology was negative, and pelvic lymph node dissection showed no metastatic involvement (0/13). No gross extra-ovarian disease was identified.

### 2.4 Pathological and immunohistochemical findings

Gross examination revealed a left adnexal tumor measuring approximately 12 × 8 × 5 cm with a cystic-solid appearance, accompanied by extensive hemorrhage and necrosis.

Microscopically, the tumor was composed of high-grade small round cells arranged in diffuse sheets and nests. The tumor cells exhibited scant cytoplasm, hyperchromatic nuclei, nuclear molding, frequent mitotic activity, and necrosis (Figure 4A).

**Figure 4:**
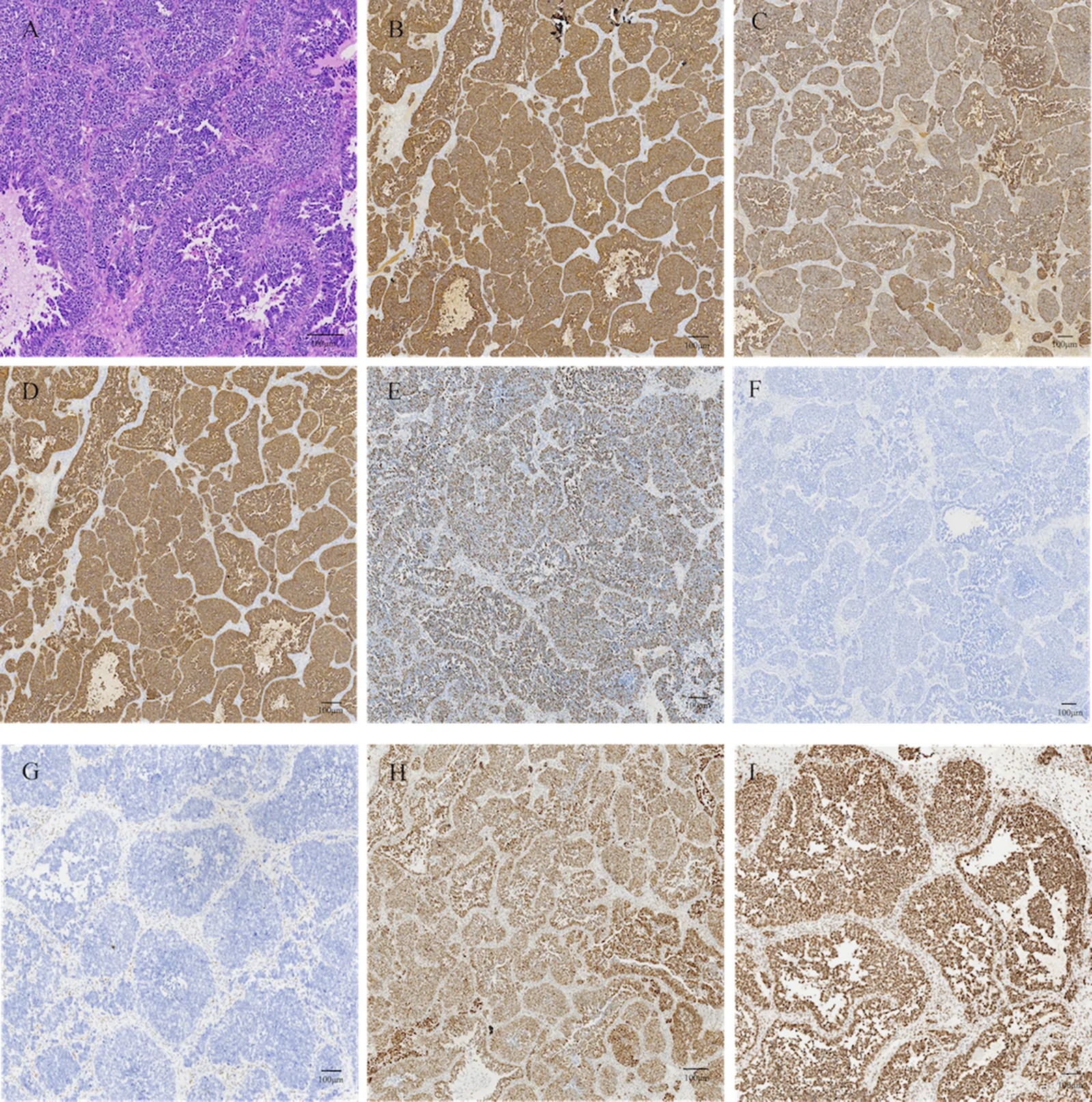
Histopathological and immunohistochemical findings of primary ovarian pulmonary-type small cell neuroendocrine carcinoma. **(A)** Hematoxylin and eosin (H&E) staining revealed diffuse sheets and nests of small round cells with scant cytoplasm, hyperchromatic nuclei, nuclear molding, frequent mitotic activity, and necrosis. **(B–D)** Immunohistochemical staining demonstrated diffuse expression of neuroendocrine markers, including synaptophysin **(B)**, chromogranin A **(C)**, and CD56 **(D) (E)** Ki-67 immunostaining showed a high proliferative index of approximately 70%. **(F)** TTF-1 immunostaining was negative in tumor cells, providing evidence against a pulmonary primary origin. **(G)** Rb immunostaining demonstrated complete loss of nuclear Rb expression in tumor cells. **(H)** p53 immunostaining showed an aberrant mutant-type overexpression pattern. **(I)** SMARCA4 immunostaining showed retained nuclear expression in tumor cells. Scale bars = 100 μm.

Immunohistochemical analysis demonstrated diffuse expression of neuroendocrine markers, including synaptophysin, chromogranin A, and CD56, with a high Ki-67 proliferation index of approximately 70% (Figures 4B–E). Focal nuclear positivity for PAX8 was identified, supporting Müllerian differentiation. TTF-1 was negative in tumor cells, arguing against a pulmonary primary origin (Figure 4F). Complete loss of Rb expression and aberrant p53 overexpression were observed. SMARCA4 immunostaining showed retained nuclear expression, helping to exclude SCCOHT (Figures 4G–I).

Taken together, the morphological, immunohistochemical, and clinical findings supported the diagnosis of small cell carcinoma of the ovary, pulmonary type (SCCOPT).

### 2.5 Molecular findings

Paired tumor–blood next-generation sequencing revealed an HRD-negative profile, with a genomic instability score (GIS) of 37, below the predefined HRD positivity threshold of 43. No pathogenic or likely pathogenic variants were identified in BRCA1 or BRCA2.

A somatic TP53 p.M237I mutation was identified, with a variant allele frequency (VAF) of 86.78%, consistent with the mutant-type p53 overexpression observed by immunohistochemistry. In addition, a germline heterozygous MSH2 p.T933I variant was detected and classified as a variant of uncertain significance (VUS). No pathogenic or likely pathogenic germline variants were identified.

The sequencing panel included MMR-related genes; however, the analysis was limited to DNA-level alterations. Mismatch repair (MMR) protein immunohistochemistry and microsatellite instability (MSI) testing were not performed; therefore, the functional significance of the MSH2 p.T933I variant remains uncertain.

### 2.6 Postoperative workup for exclusion of extra-ovarian primary tumors

Postoperative ^18F-FDG PET-CT was performed on February 12, 2026, to evaluate the possibility of an extra-ovarian primary tumor. No definite extra-ovarian primary lesion was identified. Mild FDG uptake (SUVmax 1.55) was observed in the gastric pylorus, whereas a 3-mm right upper lobe pulmonary nodule showed no abnormal FDG uptake.

Subsequent gastroscopy and colonoscopy were performed on March 19, 2026. Gastric biopsy demonstrated chronic inflammatory changes and benign fundic gland polyps without evidence of malignancy. Colonoscopic examination was unremarkable.

Overall, these investigations identified no evidence of an extra-ovarian primary tumor. Although an occult primary site cannot be completely excluded, the comprehensive postoperative evaluation, together with the clinical, pathological, immunohistochemical, and molecular findings, strongly supported the diagnosis of primary ovarian pulmonary-type small cell neuroendocrine carcinoma (SCCOPT).

### 2.7 Postoperative course and oncologic management

The postoperative course was uneventful. Following multidisciplinary team discussion, adjuvant systemic chemotherapy was initiated with carboplatin (AUC 5, day 1) and etoposide (100 mg/m^2^, days 1–3), administered every three weeks.

At the latest follow-up (June 2026), the patient had completed six cycles of chemotherapy without experiencing grade ≥3 treatment-related adverse events. Contrast-enhanced abdominal CT performed before the sixth cycle (June 11, 2026) demonstrated postoperative changes at the ovarian resection site without abnormal enhancement and no radiological evidence of local recurrence or distant metastasis. The patient remained alive without clinically evident disease recurrence at the latest follow-up.

## 3 Discussion

Small cell carcinoma of the ovary, pulmonary type (SCCOPT), is an exceptionally rare and aggressive ovarian neuroendocrine carcinoma, for which clinical experience and comprehensive molecular characterization remain limited (1, 14). In a population-based study of 166 patients with ovarian neuroendocrine carcinoma, Zhu et al. reported aggressive clinical behavior and poor survival outcomes compared with most epithelial ovarian cancer subtypes (15). We report a case of primary ovarian SCCOPT presenting as acute abdomen caused by adnexal torsion, preoperative tumor rupture, and hemoperitoneum, thereby mimicking a benign gynecological emergency. Although adnexal torsion and spontaneous tumor rupture have been reported in ovarian neoplasms, the simultaneous occurrence of adnexal torsion, tumor rupture, and hemoperitoneum is uncommon in malignant ovarian tumors and has rarely been described in SCCOPT. Currently, no well-established risk factors have been identified for SCCOPT. Advanced age and postmenopausal status are frequently reported characteristics, although their role as true risk factors remains uncertain (4, 7). Unlike some ovarian malignancies with known hereditary or reproductive associations, no specific predisposing factors have been established for SCCOPT.

Due to its extreme rarity, SCCOPT has been described mainly in individual case reports and small series. Reported patients are typically older, postmenopausal women (4, 7). Histologically, SCCOPT resembles small cell lung carcinoma, showing small hyperchromatic cells, nuclear molding, frequent mitotic activity, and necrosis (8). Immunohistochemically, neuroendocrine markers such as synaptophysin, chromogranin A, and CD56 are commonly expressed, whereas exclusion of metastatic extra-ovarian neuroendocrine carcinoma remains essential (16). Molecular data remain limited, and current treatment strategies are largely based on surgical resection followed by platinum-based chemotherapy; however, optimal management and prognostic factors remain unclear (1–3). A comparison of the clinicopathological, molecular, therapeutic, and prognostic features of previously reported cases and our patient is summarized in Table 1.

**Table 1:** Clinicopathological, molecular, therapeutic, and prognostic features of previously reported SCCOPT cases and the present case.

**Feature**	**Previously reported SCCOPT cases**	**Present case**
Age and menopausal status	Most reported cases occurred in postmenopausal women.	61-year-old postmenopausal woman.
Clinical presentation	Common symptoms include abdominal pain, pelvic mass, abdominal distension, or abnormal vaginal bleeding. Presentation as acute abdomen has rarely been described.	Acute abdomen caused by adnexal torsion, tumor rupture, and hemoperitoneum.
Adnexal torsion, tumor rupture, and hemoperitoneum	Rarely reported in combination.	Concurrent adnexal torsion, tumor rupture, and hemoperitoneum.
Tumor characteristics	Usually presents as a unilateral large solid or solid–cystic ovarian mass.	Left unilateral solid–cystic ovarian mass with hemorrhage and necrosis.
Histopathological features	Small round to oval cells arranged in sheets or nests with nuclear molding, frequent mitoses, and extensive necrosis.	Typical small-cell morphology with nuclear molding, brisk mitotic activity, extensive necrosis, and lymphovascular invasion.
Immunophenotype	Positive for Syn, CgA, and CD56; PAX8 may be focally positive, whereas TTF-1 is usually negative.	Syn+, CgA+, CD56+, Ki-67≈70%, p53 overexpression, focal PAX8+, SMARCA4 retained, TTF-1−, and complete loss of Rb expression.
Differential diagnosis	Metastatic small cell lung carcinoma, other high-grade neuroendocrine carcinomas, and SCCOHT should be excluded.	Diagnosis was established based on clinicopathological findings, immunophenotype, molecular testing, PET-CT, and gastrointestinal endoscopy.
Molecular characteristics	Only a limited number of reported cases have undergone molecular testing.	TP53 p.M237I mutation, complete loss of Rb expression, HRD-negative status, BRCA1/2 wild type, and germline MSH2 p.T933I (VUS).
Treatment	Cytoreductive surgery followed by platinum-based chemotherapy is the most commonly reported treatment.	Comprehensive staging surgery followed by six cycles of carboplatin plus etoposide.
Outcome/prognosis	Reported outcomes suggest an overall poor prognosis, although long-term follow-up is limited.	No evidence of recurrence or metastasis during the current follow-up.

SCCOPT, small cell carcinoma of the ovary, pulmonary type; SCCOHT, small cell carcinoma of the ovary, hypercalcemic type; HRD, homologous recombination deficiency; VUS, variant of uncertain significance.

Clinical, pathological, therapeutic, and prognostic information was extracted from published SCCOPT case reports, case series, and reviews, while molecular findings were supplemented by available molecular studies (4, 7, 8, 12, 13, 16, 21).

Malignant ovarian tumors are generally considered less susceptible to torsion because infiltrative growth, pelvic adhesions, and reduced tumor mobility may limit adnexal rotation. Adnexal torsion is more frequently associated with benign ovarian lesions; however, torsion in postmenopausal women remains clinically important because malignant pathology, although uncommon, may occasionally be identified in this population (17). Consequently, when torsion is accompanied by hemorrhagic changes and acute abdominal symptoms, the underlying malignant nature of the lesion may be obscured by an apparently benign emergency presentation. Furthermore, the imaging manifestations of adnexal torsion are variable and may overlap with those of other complex adnexal lesions, contributing to preoperative diagnostic uncertainty (18). In the present case, although preoperative MRI demonstrated a complex cystic-solid adnexal mass with suspicious enhancing solid components, the coexistence of torsion, tumor rupture, and hemoperitoneum initially favored the diagnosis of a benign adnexal emergency rather than an aggressive ovarian malignancy. This case highlights the importance of maintaining ovarian malignancy in the differential diagnosis of postmenopausal women presenting with acute abdominal pain, adnexal masses, and pelvic free fluid, even when the initial clinical and radiological impression favors benign adnexal torsion.

The major diagnostic challenge in this case was establishing the primary ovarian origin of the neuroendocrine carcinoma. Histologically, SCCOPT shares substantial morphological overlap with small cell lung carcinoma (SCLC) and other high-grade neuroendocrine carcinomas; therefore, exclusion of metastatic disease is essential before assigning an ovarian primary diagnosis (7, 8, 16). In our patient, the diagnosis was established through integration of the clinical presentation, radiological findings, histopathological features, immunophenotype, molecular profile, and comprehensive postoperative investigations.

Histopathologically, the tumor exhibited classic high-grade small-cell features, including diffuse sheets and nests of tumor cells, nuclear molding, frequent mitotic activity, and extensive necrosis. Immunohistochemically, diffuse expression of synaptophysin, chromogranin A, and CD56 confirmed neuroendocrine differentiation, while the high Ki-67 proliferation index supported the highly proliferative nature of the tumor. Focal PAX8 expression supported Müllerian differentiation, a finding compatible with an ovarian primary in the appropriate clinicopathological context (19). In contrast, negative TTF-1 expression, together with the absence of a detectable pulmonary primary lesion on postoperative PET–CT, argued against metastatic SCLC, although neither marker alone is sufficient to determine the site of origin. Although focal CK20 and SATB2 expression raised consideration of a gastrointestinal primary tumor, subsequent gastroscopy, colonoscopy, and imaging evaluation revealed no evidence of an extra-ovarian primary malignancy. Furthermore, retained SMARCA4 expression helped distinguish this tumor from small cell carcinoma of the ovary, hypercalcemic type (SCCOHT), in which SMARCA4 inactivation is characteristic (6, 20). Taken together, these clinicopathological, immunophenotypic, and radiological findings strongly supported the diagnosis of primary ovarian SCCOPT. The comprehensive postoperative evaluation—including PET–CT, gastroscopy, and colonoscopy—further supported an ovarian primary origin by excluding clinically detectable pulmonary, gastrointestinal, and other common primary sites, although an occult extra-ovarian primary cannot be completely excluded.

The genomic architecture of SCCOPT remains largely uncharacterized, with comprehensive molecular profiling reported in only a limited number of cases (1, 2). Recent molecular data suggest that SCCOPT shares some biological features with small cell lung carcinoma, although the basis of these similarities remains unclear (21). Molecular testing may complement histopathological and immunohistochemical evaluation by providing additional insights into diagnostic assessment, molecular characterization, and treatment considerations. In particular, SMARCA4 status may provide additional support in distinguishing SCCOPT from SCCOHT, which is characterized by SMARCA4 inactivation (5, 6). TP53 pathway alterations have been described in ovarian pulmonary-type neuroendocrine carcinomas, although their biological and prognostic significance remains uncertain (1, 7). In our patient, paired tumor–blood next-generation sequencing demonstrated an HRD-negative, BRCA1/2 wild-type profile. The principal somatic alteration was a TP53 p.M237I mutation with a high variant allele frequency, which correlated with the mutant-type p53 overexpression observed by immunohistochemistry. Complete loss of Rb expression was also identified. This concurrent disruption of TP53 and Rb, a molecular feature frequently observed in SCLC, may partly contribute to the aggressive biological behavior of SCCOPT (22). However, whether these alterations have independent prognostic significance remains unknown and will require additional molecularly characterized cases with long-term follow-up.

Because SCCOPT is exceedingly rare, treatment recommendations remain largely empirical, and no standard therapeutic strategy has been established (1, 3). Current management is generally extrapolated from SCLC and other high-grade neuroendocrine carcinomas, with complete surgical resection followed by platinum-based chemotherapy representing the most widely adopted approach (1, 3). Following intraoperative suspicion of malignancy, our patient underwent comprehensive surgical staging and subsequently received six cycles of carboplatin plus etoposide. Treatment was well tolerated, with no grade 3 or higher adverse events, and follow-up imaging demonstrated no evidence of recurrence or distant metastasis. Carboplatin was selected instead of cisplatin to improve postoperative tolerability while preserving the established platinum–etoposide backbone. In this patient, the HRD-negative and BRCA1/2 wild-type profile did not provide a molecular rationale for considering PARP inhibitor maintenance therapy.

Several limitations should be acknowledged. This report describes a single patient with relatively short follow-up, precluding any firm conclusions regarding long-term prognosis. Next-generation sequencing identified a germline MSH2 variant of uncertain significance. However, because MMR immunohistochemistry and MSI testing were not performed, the clinical relevance of this variant cannot be fully determined. Nevertheless, this case contributes additional clinicopathological and molecular evidence to the limited literature on SCCOPT and underscores an important diagnostic pitfall: this rare tumor may present as a benign-appearing surgical emergency.

This case underscores that ovarian malignancy should remain in the differential diagnosis of postmenopausal women presenting with acute abdominal pain, an adnexal mass, and pelvic free fluid, even when the initial clinical impression favors benign adnexal torsion. Accurate diagnosis of SCCOPT requires integration of the clinical presentation, imaging findings, histopathology, immunophenotype, molecular profile, and systematic exclusion of an extra-ovarian primary tumor. Increased awareness of this diagnostic pitfall may facilitate earlier recognition of this rare malignancy in emergency settings.

## 4 Conclusion

We report a rare case of primary ovarian SCCOPT presenting with acute pelvic pain, adnexal torsion, tumor rupture, and hemoperitoneum, mimicking a benign gynecological emergency. This case highlights that malignancy should remain an important consideration in postmenopausal women with adnexal masses and acute abdominal symptoms, even when torsion is initially suspected.

Accurate diagnosis requires an integrated approach combining imaging, histopathology, immunohistochemistry, molecular profiling, and comprehensive exclusion of extra-ovarian primary tumors. Given the extreme rarity of SCCOPT, standardized treatment strategies have not yet been established. Additional well-characterized cases with long-term follow-up are needed to better define its biological behavior and guide clinical management.

## Data Availability

The datasets presented in this article are not readily available because they contain potentially identifiable patient information and clinical sequencing data from a single case. The data may be made available by the corresponding author upon reasonable request, subject to applicable ethical and institutional requirements. No public accession number is available for the sequencing data because the patient-level clinical sequencing data have not been deposited in a public repository due to privacy and ethical considerations. Requests to access the datasets should be directed to the corresponding author.

## References

[1] Di Vito V Veroi G Rizza L Rota F Baiocchini A Macciomei MC. Ovarian neuroendocrine neoplasms: challenges and future perspectives. J Clin Med. (2025) 14:8833. 10.3390/jcm14248833 41464736 PMC12733891

[2] Zhao Y Kang F Kong X Wang N. A case report of small cell neuroendocrine carcinoma of the ovary and review of the literature. Front Immunol. (2025) 16:1569011. 10.3389/fimmu.2025.1569011 40475766 PMC12137309

[3] He Q Wang C Huang D Shen J Liu R Guan Y. Clinicopathologic features and treatment progress of high-grade ovarian neuroendocrine tumors. Med Oncol. (2024) 42:2. 10.1007/s12032-024-02544-w 39541042

[4] Kalampokas E Sharma V Gagliardi T Payne F Gurumurthy M. Primary small cell ovarian cancer of pulmonary type: a case report. Gynecol Oncol Rep. (2018) 24:10–3. 10.1016/j.gore.2018.02.003 29552627 PMC5852300

[5] WHO Classification of Tumours Editorial Board. Female Genital Tumours. 5th ed. Lyon (France): International Agency for Research on Cancer; 2020.

[6] Witkowski L Carrot-Zhang J Albrecht S Fahiminiya S Hamel N Tomiak E. Germline and somatic SMARCA4 mutations characterize small cell carcinoma of the ovary, hypercalcemic type. Nat Genet. (2014) 46:438–43. 10.1038/ng.2931 24658002

[7] Chen X Liu HL Wang JS Zhao FH. Primary ovarian small cell carcinoma of pulmonary type: analysis of 6 cases and review of 31 cases in the literature. Chin Med Sci J. (2023) 38:130–7. 10.24920/004183 37286512

[8] Gupta P Kapatia G Gupta N Dey P Rohilla M Gupta A. Small cell carcinoma of the ovary: clinicopathologic and immunohistochemical analysis of 7 new cases of a rare malignancy. Int J Surg Pathol. (2021) 29:236–45. 10.1177/1066896920947788 32772748

[9] Conlon N Silva A Guerra E Jelinic P Schlappe BA Olvera N. Loss of SMARCA4 expression is both sensitive and specific for the diagnosis of small cell carcinoma of ovary, hypercalcemic type. Am J Surg Pathol. (2016) 40:395–403. 10.1097/PAS.0000000000000558 26645725 PMC4752399

[10] Li M Wang H Hou S Wang S Li H. Comparison of characteristics and outcomes of premenopausal and postmenopausal women with adnexal torsion. J Int Med Res. (2024) 52:3000605241305251. 10.1177/03000605241305251 39711154 PMC11672383

[11] Bailey F Moore G De A Holland T. Ovarian torsion: a modern approach to management. Obstet Gynaecol. (2025) 27:221–30. 10.1111/tog.12985

[12] Suzuki N Kameyama K Hirao T Susumu N Mukai M Aoki D. A case of pulmonary type of ovarian small cell carcinoma. J Obstet Gynaecol Res. (2007) 33:203–6. 10.1111/j.1447-0756.2007.00494.x 17441897

[13] Najib FS Momtahan M Mokhtari M M Alamdarloo S Poordast T Small cell carcinoma of the ovary: report of a case with unusual and aggressive presentation. Middle East J Cancer. (2017) 8:161–5.

[14] Čelebić A Harasani K Miladinović M Mandić A Vasiljević T Drusany Starič K. Neuroendocrine tumors of the gynecological tract: a narrative literature review. Eur J Surg Oncol. (2025) 51:109735. 10.1016/j.ejso.2025.109735 40057385

[15] Zhu Y Meng F Fang H Zhang Z Wang L Zheng W. Clinicopathologic characteristics and survival outcomes in neuroendocrine carcinoma of the ovary. Int J Gynecol Cancer. (2020) 30:207–12. 10.1136/ijgc-2019-000746 31796530

[16] Oneda E Zorzi F Gorio A Quaglia F Abeni C Rota L. Differential diagnosis of small cell carcinoma of the ovary or ovarian metastases of small cell carcinoma of the lung: a case report and review of the literature. Case Rep Oncol. (2020) 13:822–8. 10.1159/000507978 32884525 PMC7443635

[17] Ganer Herman H Shalev A Ginath S Kerner R Keidar R Bar J. Clinical characteristics and the risk for malignancy in postmenopausal women with asdnexal torsion. Maturitas. (2015) 81:57–61. 10.1016/j.maturitas.2015.02.261 25804950

[18] Dawood MT Naik M Bharwani N Sudderuddin SA Rockall AG Stewart VR. Adnexal torsion: review of radiologic appearances. Radiographics. (2021) 41:609–24. 10.1148/rg.2021200118 33577417

[19] Odóñez NG. Value of PAX8 immunostaining in tumor diagnosis: a review and update. Adv Anat Pathol. (2012) 19:140–51. 10.1097/PAP.0b013e318253465d 22498579

[20] Jelinic P Mueller JJ Olvera N Dao F Scott SN Shah R. Recurrent SMARCA4 mutations in small cell carcinoma of the ovary. Nat Genet. (2014) 46:424–6. 10.1038/ng.2922 24658004 PMC5699446

[21] Miglietta F. MicroRNA and metabolic profiling of a primary ovarian neuroendocrine carcinoma pulmonary-type reveals a high degree of similarity with small cell lung cancer. Noncoding RNA. (2022) 8:69. 36287116 10.3390/ncrna8050064 PMC9611163

[22] George J Lim JS Jang SJ Cun Y Ozretić L Kong G. Comprehensive genomic profiles of small cell lung cancer. Nature. (2015) 524:47–53. 10.1038/nature14664 26168399 PMC4861069

