# Supplementary material for: Case Report: Primary ovarian pulmonary-type small cell neuroendocrine carcinoma mimicking benign adnexal torsion: a diagnostic challenge
Source: Front Med (Lausanne). 2026 Sep 14;13:1941804. doi: 10.3389/fmed.2026.1941804 (PMC13617120; doi:10.3389/fmed.2026.1941804)
Supplement: Supplementary file 1 [file Supplementaryfile1.docx]

**CARE Checklist for the case report**

| **CARE Checklist Item** | **Description** | **Location in Manuscript** |
| --- | --- | --- |
| Title | Primary ovarian pulmonary-type small cell neuroendocrine carcinoma mimicking benign adnexal torsion: a diagnostic challenge | Title page |
| Key words | Small cell carcinoma of the ovary, pulmonary type; acute abdomen; adnexal torsion; hemoperitoneum; neuroendocrine carcinoma | Keywords |
| Abstract | Background, case presentation, diagnostic findings, treatment, follow-up, and conclusion are presented | Abstract |
| Introduction | Background information, rarity, clinical significance, diagnostic challenges, and rationale for reporting this case are provided | Introduction |
| Patient information | Patient demographic characteristics, medical history, presenting symptoms, and relevant clinical information are described | Case Presentation – Patient History and Clinical Findings |
| Clinical findings | Physical examination findings, laboratory results, and initial clinical findings are reported | Case Presentation – Patient History and Clinical Findings; Laboratory and Imaging Findings |
| Timeline | Chronological sequence of clinical events from initial presentation, diagnosis, treatment, and follow-up | Figure 1. Clinical timeline |
| Diagnostic assessment | Diagnostic evaluation including imaging studies, histopathology, immunohistochemistry, molecular profiling, and exclusion of extra-ovarian primary tumors is described | Laboratory and Imaging Findings; Pathological and Immunohistochemical Findings; Molecular Findings; Postoperative Workup for Exclusion of Extra-ovarian Primary Tumors |
| Differential diagnosis | Differential diagnoses including metastatic small cell lung carcinoma, gastrointestinal neuroendocrine carcinoma, and small cell carcinoma of the ovary, hypercalcemic type are discussed | Pathological and Immunohistochemical Findings; Discussion |
| Therapeutic intervention | Surgical management, staging procedure, chemotherapy regimen, and treatment rationale are described | Surgical Findings and Management; Postoperative Course and Oncologic Management |
| Follow-up and outcomes | Treatment response, adverse events, follow-up imaging findings, and current disease status are reported | Postoperative Course and Oncologic Management |
| Discussion | Interpretation of findings, comparison with previous literature, diagnostic challenges, molecular characteristics, treatment considerations, and limitations are discussed | Discussion |
| Patient perspective | Patient perspective was not available | Not available |
| Informed consent | Written informed consent for participation and publication of this case report was obtained from the patient | Ethics statement |
