# Supplementary material for: Case Report: Primary ovarian pulmonary-type small cell neuroendocrine carcinoma mimicking benign adnexal torsion: a diagnostic challenge
Source: Front Med (Lausanne). 2026 Sep 14;13:1941804. doi: 10.3389/fmed.2026.1941804 (PMC13617120; doi:10.3389/fmed.2026.1941804)
Supplement: Supplementary file 2 [file Table1.docx]

**Supplementary Table S1.** Immunohistochemical profile and diagnostic evaluation of ovarian SCCOPT

| **Marker** | **Result** | **Interpretation** |
| --- | --- | --- |
| **Neuroendocrine markers** | | |
| Synaptophysin | Positive (+) | Supports neuroendocrine differentiation |
| Chromogranin A | Positive (+) | Supports neuroendocrine differentiation |
| CD56 | Positive (+) | Supports neuroendocrine differentiation |
| **Lineage markers** | | |
| PAX8 | Focal positive | Supports Müllerian/ovarian differentiation |
| TTF-1 | Negative | Argues against metastatic pulmonary small cell carcinoma |
| Napsin A | Negative | Does not support lung adenocarcinoma differentiation |
| WT-1 | Negative | Does not support typical high-grade serous carcinoma |
| GATA3 | Negative | Does not support breast/urothelial origin |
| CEA | Negative | Does not support conventional gastrointestinal adenocarcinoma |
| Vimentin | Negative | Non-specific; not supportive of mesenchymal differentiation |
| HNF-1β | Negative | Does not support clear cell carcinoma |
| NUT | Negative | Does not support NUT carcinoma |
| CK20 | Partially positive | Non-defining; may raise consideration of gastrointestinal origin |
| SATB2 | Focal positive | Non-defining; may raise consideration of lower gastrointestinal origin |
| CD7 | Partially positive | Ancillary finding; not diagnostic |
| **Tumor suppressors / chromatin remodeler** | | |
| SMARCA4 (BRG1) | Retained | Argues against SCCOHT |
| p53 | Diffuse strong overexpression | Mutant-type pattern |
| Rb | Lost | Supports high-grade small cell neuroendocrine phenotype |
| **Proliferation marker** | | |
| Ki-67 | Approximately 70% | High proliferative activity |
