# Supplementary material for: Case Report: Primary ovarian pulmonary-type small cell neuroendocrine carcinoma mimicking benign adnexal torsion: a diagnostic challenge
Source: Front Med (Lausanne). 2026 Sep 14;13:1941804. doi: 10.3389/fmed.2026.1941804 (PMC13617120; doi:10.3389/fmed.2026.1941804)
Supplement: Supplementary file 3 [file Table2.docx]

**Supplementary Table S2.** Molecular findings of primary ovarian pulmonary-type small cell neuroendocrine carcinoma

| Category | Result |
| --- | --- |
| HRD status | Negative |
| Genomic instability score (GIS) | 37 |
| HRD positivity threshold | 43 |
| BRCA1 | No pathogenic or likely pathogenic variant detected |
| BRCA2 | No pathogenic or likely pathogenic variant detected |
| Somatic alteration | TP53 c.711G>A (p.M237I), exon 7, VAF 86.78% |
| Germline finding | MSH2 c.2798C>T (p.T933I), Variant of uncertain significance (VUS) |
| MSI/MMR evaluation | Not performed (DNA-level sequencing only) |
